# Self-perceived health in functionally independent older people: associated factors

**DOI:** 10.1186/s12877-016-0239-9

**Published:** 2016-03-09

**Authors:** Mónica Machón, Itziar Vergara, Miren Dorronsoro, Kalliopi Vrotsou, Isabel Larrañaga

**Affiliations:** Unidad de Investigación de Atención Primaria-OSIs Gipuzkoa, Osakidetza, San Sebastián, Spain; Red de Investigación en Servicios de Salud en Enfermedades Crónicas (REDISSEC), Madrid, Spain; Instituto de Investigación Sanitaria Biodonostia, San Sebastián, Spain; Dirección de Salud Pública y Adicciones, Gobierno Vasco, Vitoria, Spain; CIBER Epidemiología y Salud Pública (CIBERESP), Madrid, Spain; Departamento de Salud, Delegación Territorial de Gipuzkoa, Gobierno Vasco, San Sebastián, Spain

**Keywords:** Older adults, Self-perceived health, Ageing, Living conditions

## Abstract

**Background:**

Self-perceived health (SPH) is a powerful indicator of the health status of elderly people. This issue has been widely studied in oldest populations considering altogether functionally independent and dependent individuals. The objective of this study was to describe SPH and to identify the main factors that have an impact on SPH in a sample of functionally independent community-dwelling older adults.

**Methods:**

For this cross-sectional study, face-to-face interviews were carried out with non-institutionalized functionally independent older individuals in a northern region of Spain. Participants were asked: “Overall, you would say that your health is excellent, very good, good, fair or poor?”. SPH responses were grouped in two categories: good and poor. Binary logistic regression was used to identify factors associated with poor SPH.

**Results:**

A sample of 634 individuals was studied, of whom 55 % were women. The mean age was 74.8 (SD 6.7) years. About 18 % of the respondents rated their health as poor. In the multivariate model adjusted for age and sex, reported poor health was significantly associated with polypharmacy (≥3 drugs per day) (OR: 5.76, 95 % CI: 3.60–9.18), the presence of sensory impairment (OR: 1.87, 95 % CI: 1.15–3.04), bad sleep quality (OR:1.82, 95 % CI: 1.02–3.28), a bad nutrition pattern (OR: 2.37, 95 % CI: 1.08–5.21), not engaging in cognitively stimulating activities (OR: 4.08, 95 % CI: 1.64–10.20), or group social activities (OR: 2.62, 95 % CI: 1.63–4.23).

**Conclusions:**

The study indicates that several health and social variables are strongly related to SPH in independent community-dwelling older adults. This finding highlights the need for thorough assessment of factors related to SPH in older independent adults, this being essential to develop health-related programmes for promoting active and healthy ageing and to delay the onset of dependence in this population.

## Background

Population ageing is a worldwide trend in recent decades. In Spain, there were an estimated 7.9 million people (17 % of the total population) over the age of 65 in 2014 and this figure is expected to reach 15.4 million (38 % of the total population) by 2064 [1]. Active and healthy ageing (AHA) is a multi-factorial process that has become a health policy priority for local, national and international health authorities [2] in order to reduce the growing incidence of dependence. Self-perceived health (SPH) is included in one of the six group of determinants of the AHA model proposed by the World Health Organization (WHO) [3].

SPH is a complex measure that “represents a summary statement about the way in which numerous aspects of health, both subjective and objective, are combined within the perceptual framework of the individual respondent” [4]. It has been widely used, and is considered a valid and reliable indicator [5–7] of overall health status, a predictor of mortality and of health services use [8]. It is usually evaluated with a single item, asking individuals to rate their overall health on a scale from poor to excellent. Several alternative phrasings of the SPH question exist, but all have similar response patterns [9].

SPH in older adults has been found to be associated to sociodemographic characteristics (namely, sex, age, education, and income), chronic diseases and functional status [10–12]. Additional factors linked to SPH are social relationships [11–13] and neighborhood environment [14, 15]. However, the effect magnitude of these factors varies depending on the study design, population and cultural context [16], and not all studies include all the above mentioned variables.

Functional status, in particular, is widely recognised to be a powerful determinant of SPH in older adults [10]. The assessment of functional status is a key element in healthcare. The detection of functionality loss can guide the implementation of health interventions aimed to avoid or delay the onset of dependence. Given the relevance of functional status as a factor of SPH, when it is studied at the same time with other related factors, the latter may become secondary with their role remaining undervalued when functional status is considered. Possibly, this is why less is known about how older people with good functional status perceive their health and the actual role of other factors related to SPH in this population. The assessment of factors beyond functional status may provide relevant information for the design and implementation of health promotion strategies.

This work is part of a larger study focused on understanding aspects of health and living conditions of community-dwelling elderly people, from a comprehensive perspective. The objectives of this study are to describe SPH in a functionally independent community-dwelling older population and to identify the main factors associated with SPH in this population group.

## Methods

### Sample size

A sample of 800 older individuals, from the province of Gipuzkoa (with a population of over 708,000 [17]) in Spain, was randomly selected using multistage sampling. The derived sample was representative of the Gipuzkoa elderly population in terms of age (65–74, 75–84 and ≥85 years) and sex according to the Basque Health Survey data [18].

### Fieldwork process

The data collection took place between January 2013 and February 2013. Trained interviewers conducted face-to-face interviews with the selected individuals in their homes. The information was collected using a battery of 145 questions that explored health and living conditions (Table 1). It was constructed based on the published literature and the recommendations of a multidisciplinary expert panel. A partner, relative, friend or caregiver was allowed to be present during the interview if so desired by the participants to feel more confident. This did not interfere with the subjective questions (e.g., SPH), that should be answered only by the older person.

**Table 1:** Dimensions assessed, main variables and scales administered to the participants in the full battery

Health and living condition dimensions assessed	Main variables and scales used
Sociodemographic characteristics	Age; sex; marital status; level of education; occupational status; income
Health status	Functional status (BI, and LS, respectively); self-perceived health; health-related quality of life (EQ-5D); chronic diseases; drug prescriptions; cognitive status (SPMSQ); depressive symptoms (GDS); falls; sensory impairment
Lifestyle	Smoking status; drinking habits; sleep; diet; undernutrition (STARU); physical activity
Social relationships	Leisure activities, household composition; help and care given and received; social capital (STS); social support from family and friends (LSNS); functional social support (Duke-UNC FSSQ)
Social services	Use of social services
Health services	Use of health services
Housing	Housing tenure; facilities (lift, shower/bath, and mobile phone, among others); housing condition; indoor and outdoor access
Neighbourhood environment	Proximity of services; adequacy of community services

*BI* Barthel index, *LS* Lawton instrumental activities of daily living scale, *EQ-5D* EuroQol 5D questionnaire, *SPMSQ* short portable mental status questionnaire of Pfeiffer, *GDS* geriatric depression scale, *STARU* screening tool for assessing risk of undernutrition, *STS* social trust scale, *LSNS* Lubben social network scale, *Duke-UNC FSSQ* Duke-UNC functional social support questionnaire

The study was approved by the *Ethics Committee of Gipuzkoa Health Region*. Written informed consent was obtained from all participants.

### Measurements

SPH was the main outcome of interest. It was measured using a single item. “Overall, you would say that your health is…”, with five response options: excellent; very good; good; fair; and poor. This item and response options have been widely used in previous research studies and health surveys [13, 14, 16, 18]. All responses were grouped into two categories, good (excellent/very good/good) and poor (fair/poor); approach adopted by other researchers [10, 19].

The following individual, social and contextual variables were assessed.

First, in relation to the individual, sociodemographic characteristics were considered: sex, age, level of education, monthly family income and living arrangements.

Regarding health, several variables were studied. Cognitive status was measured with the Pfeiffer’s Short Portable Mental Status Questionnaire [20, 21]. This test is composed of ten items and the total number of errors made in answering them is recorded. Functional status was assessed using the Barthel Index [22, 23] measuring ability to perform basic activities of daily living (BADL). This scale consists of ten items and its overall possible score ranges from 0 to 100, with lower scores indicating more severe dependence. Diagnosed chronic conditions, including diabetes, hypertension, and cardiovascular disease, among others were recorded. Moreover, participants were asked about prescription drugs taken daily for the previous 2 weeks, considering polypharmacy if they took three or more [24]. Depressive symptoms were measured with the Geriatric Depression Scale [25, 26], which contains 15 items (10 positive and 5 negative). A score of 5 or above is considered to suggest the presence of depressive symptoms. Further, the number of falls occurred in the previous 12 months was recorded. Lastly, a variable was created indicating whether participants had difficulties with sensory-related abilities, considering hearing and vision, but also speech and chewing. The latter was considered part of the sensory-related abilities due to the fact that impaired masticatory abilities may have an impact on general health and quality of life [27]. Sensory impairment was defined as having difficulties with at least one of the aforementioned abilities.

Lifestyle habits analysed included participants’ smoking status; whether they had done any physical activity in the previous 2 weeks; and the sleep quality, that is, whether they generally got a good night’s sleep, good enough to feel well rested. Furthermore, a validated screening tool was used for assessing the risk of undernutrition [28, 29]. It is composed of nine items and its total score ranges from a minimum of 0 to a maximum of 26 points. A score of 7 or above was considered to indicate that a person was at risk of being undernourished or actually undernourished presenting a bad nutrition pattern.

Second, regarding social variables, we investigated how participants spent their leisure time, asking them how frequently in the last 12 months they had engaged in the following: cognitively stimulating activities (reading, listening to the radio); active leisure activities (going for a walk, taking care of a pet, gardening); social leisure activities (spending time with friends, attending sport events, going to a dance club, going to the cinema/theatre or a concert, going to a bar or out to have lunch/dinner); and group social activities (going on holiday). For the first three, individuals were considered as attending a certain type of activity if they participated in at least one of the activities of that group.

To assess social support, we used the Lubben Social Network Scale [30, 31]. This instrument includes six items about perceived social support from family and friends, with each response rated from 0 to 5. The total score is an equally-weighted sum of scores on these six items. A higher score indicates a higher level of perceived social support. Results were dichotomized, using a cut-off point of less than 12 for the whole scale. Functional social support was measured using the 11-item Duke-UNC Functional Social Support Questionnaire [32, 33]. Responses are scored on a 5-point likert scale with higher scores indicating a higher level of social support [33]. A total score of ≤32 was considered to indicate that the respondent had a low level of functional social support. Social capital was evaluated with a three-item scale used in the *European Social Survey* [34]. All questions were answered on an 11-point scale ranging from 0 representing “low trust” to 10 representing “high trust”. In the constructed scale, total scores of up to 5 were considered to reflect low social trust and scores over 5 high social trust. Further, participants were asked to rate their social life, and responses were grouped into: satisfactory (very satisfactory/satisfactory), and unsatisfactory (unsatisfactory/very unsatisfactory).

Lastly, regarding context, participants were asked to state whether there were physical barriers or other obstacles to their mobility in their home (yes/no); and to rate the overall condition of their home, classifying this self-reported housing condition as good (good, very good or excellent) or poor (poor, fair). Community and neighbourhood resources were assessed using 21 items, based on the Checklist of Essential Features of Age-friendly Cities of the WHO [35], exploring the following features: outdoor spaces (pavements, green spaces, public toilets and streets), public buildings (elevators, toilets and ramps) and public transport (accessible vehicles, priority seats and drop-off spots). Answers were merged into two categories: none/very few/few vs. some/many/very many. Community services were considered adequate if the individual answered some/many/very many to questions about whether there were green spaces, public toilets, accessible public transport and priority seats on public transport. In addition, an item was included asking whether they walked or used transport to travel to a number of facilities.

Questions requiring a frequency response (e.g., number of prescription drugs consumed, cigarettes smoked last week) lacked close answers and were replied in a numerical form by the participants. No open reply opinion questions were asked in the study.

### Exclusion criteria

We excluded individuals with cognitive impairment, as this could compromise their ability to provide valid answers, and also those with dependence. Individuals were classified as a) having cognitive impairment, if they could read and made three or more errors, or if they were illiterate and made four or more errors, in Pfeiffer’s Short Portable Mental Status Questionnaire [20, 21]; and b) dependent, if they obtained a Barthel Index score of <95 points [22, 23].

### Statistical analysis

Categorical variables are presented as frequencies with percentages (%) and continuous variables as means with standard deviations (SD). Chi-square or Fisher’s exact tests were implemented for comparing categorical variables and Student’s *t*-test was used in two-group comparisons of continuous variables. Univariate and multivariate binary logistic regression models adjusted for age and sex were fitted for the SPH outcome. All variables with *p*-values <0.10 at the univariate stage were considered during the multivariate analysis phase. Both backward and forward regression analysis was performed. The model results are presented as odds ratios (OR) with their respective 95 % confidence intervals (95 % CI) and *p*-values. Differences were considered statistically significant if *p*-values were <0.05. The correlation matrix of estimated parameters, their eigenvalues and proportion of variation were examined for assessing collinearity presence [36]. The Hosmer-Lemeshow test and R-square and area under the curve (AUC) values are given for the final model. All analyses were performed with SAS 9.3 software.

## Results

Of the 800 individuals selected, 125 were excluded from the analysis for cognitive impairment and 41 for BADL dependence. Those excluded were mostly women (67 %), were older (*p* < 0.0001) and had poorer health, with a higher percentage having ≥3 chronic conditions (*p* < 0.0001) and taking ≥3 prescription drugs (*p* < 0.0001) compared to the included individuals. Thus, the final sample included in this study consisted of 634 independent community-dwelling older adults. The mean age of the individuals included was 74.8 (SD 6.7) years and more than half (55 %) of them were women. A total of 126 subjects were accompanied during the interview and 12 (2 %) of the participants answered the battery of questions with some help by the accompanying person. The five SPH categories were replied as follows: excellent (*n* = 38 participants), very good (*n* = 147), good (*n* = 333), fair (*n* = 105) and poor (*n* = 11). Thus, about 18 % of the respondents rated their SPH as poor.

From an individual perspective, those with a higher level of education were more likely to report a good SPH (*p* = 0.035), as were those with higher monthly incomes (*p* = 0.053) (Table 2). In addition, those presenting ≥3 chronic diseases and taking ≥3 drugs daily (*p* < 0.0001), having depressive symptoms (*p* < 0.0001), sensory impairment (*p* = 0.0001), bad nutrition pattern (*p* = 0.0001) and failing to get a good night’s sleep (*p* = 0.002) were more likely to report a poor SPH.

**Table 2:** Characteristics of older people according to their self-perceived health

	Self-perceived health		
Variables	Poor (*n* = 116)	Good (*n* = 518)	*p*-value
Sex			
Men	47 (16)	238 (84)	0.288
Women	69 (20)	280 (80)	
Age (years), mean (SD)	74.9 (6.3)	74.8 (6.8)	0.865
Level of education			
Primary or lower	103 (20)	416 (80)	0.035
Secondary or higher	13 (11)	101 (89)	
Monthly family income (€)			
≤1500	68 (20)	277 (80)	0.053
≥1501	18 (12)	127 (88)	
Living arrangements			
Alone	29 (18)	130 (82)	0.974
With others	87 (18)	387 (82)	
Number of diagnosed chronic diseases			
0–2	45 (10)	403 (90)	<0.0001
≥3	71 (38)	115 (62)	
Number of drugs taken daily			
0–2	38 (9)	384 (91)	<0.0001
≥3	78 (37)	134 (63)	
GDS score			
Not depressive symptoms (<5)	86 (15)	470 (85)	<0.0001
Depressive symptoms (≥5)	30 (38)	48 (62)	
Falls in the previous 12 months			
0	91 (18)	424 (82)	0.396
≥1	25 (21)	94 (79)	
Sensory impairment			
≥1	46 (28)	117 (72)	0.0001
0	70 (15)	401 (85)	
Smoker			
Yes	7 (17)	35 (83)	0.774
No	109 (18)	482 (82)	
Physical activity in the previous 2 weeks			
Yes	94 (18)	437 (82)	0.380
No	22 (21)	81 (79)	
Sleep quality			
Good	89 (16)	463 (84)	0.002
Bad	27 (33)	55 (67)	
Nutrition pattern (STARU)			
Good (<7)	101 (17)	498 (83)	0.0001
Bad (≥7)	15 (43)	20 (57)	
Cognitively stimulating activities			
Yes	104 (17)	504 (83)	<0.0001
No	12 (46)	14 (54)	
Active leisure activities			
Yes	110 (18)	503 (82)	0.245
No	6 (29)	15 (71)	
Social leisure activities			
Yes	90 (16)	462 (84)	0.001
No	26 (32)	56 (68)	
Group social activities			
Yes	41 (12)	305 (88)	<0.0001
No	75 (26)	211 (74)	
LSNS score			
Low level of perceived social support (<12)	27 (19)	113 (81)	0.732
High level of perceived social support (≥12)	89 (18)	405 (82)	
Duke-UNC FSSQ score			
Low level of functional social support (≤32)	11 (15)	61 (85)	0.416
High level of functional social support (>32)	105 (19)	457 (81)	
STS			
Low social trust (≤5)	49 (24)	159 (76)	0.017
High social trust (>5)	67 (16)	359 (84)	
Self-perceived social life			
Un*s*atisfactory	18 (42)	25 (58)	<0.0001
Satisfactory	98 (17)	493 (83)	
Obstacles inside the home			
Yes	15 (29)	36 (71)	0.031
No	100 (17)	480 (83)	
Self perceived housing condition			
Good	101 (17)	477 (83)	0.085
Poor	15 (27)	41 (73)	
Adequate community services			
Yes	15 (14)	95 (86)	0.164
No	101 (19)	423 (81)	
Supermarket within walking distance			
Yes	83 (16)	432 (84)	0.003
No	33 (28)	86 (72)	

Numbers are n (%) unless otherwise stated. Row percentages are presented. When missing data exist, frequencies do not add up to column totals. *GDS* geriatric depression scale, *STARU* screening tool for assessing risk of undernutrition, *LSNS* Lubben social network scale, *Duke-UNC FSSQ* Duke-UNC functional social support questionnaire, *STS* social trust scale

From a social point of view, the percentage of those reporting good health was significantly higher among those who engaged in cognitively stimulating activities (*p* < 0.0001), social leisure activities (*p* = 0.001) or group social activities (*p* < 0.0001), with high social trust (*p* = 0.017) and satisfactory self-perceived social life (*p* < 0.0001) than it was for those who did not engage in the social activities considered, had low social trust or unsatisfactory social life.

Regarding contextual variables, individuals without obstacles inside their home (*p* = 0.031) and with a supermarket within walking distance (*p* = 0.003) were more likely to perceive their health as good.

No statistically significant differences were observed by age or sex, living arrangements, history of falls, social support, physical activity, smoking status, housing condition, active leisure activities or adequacy of community services (Table 2).

At the multivariate analysis stage, polypharmacy, sensory impairment, bad sleep quality, bad nutrition pattern and not engaging in certain types of leisure activities were identified as the set of factors that best explained SPH. Specifically, according to this model (Table 3), respondents with polypharmacy were more likely to report poor health (OR: 5.76, 95 % CI: 3.60–9.18). Further, respondents with at least one sensory impairment, those with bad sleep quality and those with bad nutrition pattern were around twice likely to rate their SPH as poor (OR: 1.87, 95 % CI: 1.15–3.04; OR: 1.82, 95 % CI: 1.02–3.28; and OR: 2.37, 95 % CI: 1.08–5.21, respectively). Finally, individuals who did not engage in cognitively stimulating activities or group social activities were also more likely to rate their SPH as poor (OR: 4.08, 95 % CI: 1.64–10.20 and OR: 2.62, 95 % CI: 1.63–4.23, respectively). Collinearity diagnostics did not reveal any problems with the included variables. The AUC of this model was 0.810, suggesting good discriminatory capacity [37].

**Table 3:** Factors associated with poor self-perceived health in older people. Multivariate logistic regression model results

Variables	OR (95 % CI)	*p*-value
Age	0.96 (0.93–0.99)	0.037
Sex		
Women	1.00	0.979
Men	0.99 (0.62–1.58)	
Number of drugs taken daily		<0.0001
0–2	1.00	
≥3	5.76 (3.60–9.18)	
Sensory impairment		
0	1.00	0.019
≥1	1.87 (1.15–3.04)	
Sleep quality		
Good	1.00	0.045
Bad	1.82 (1.02–3.28)	
Nutrition pattern (STARU)		
Good	1.00	0.032
Bad	2.37 (1.08–5.21)	
Cognitively stimulating activities		
Yes	1.00	0.003
No	4.08 (1.64–10.20)	
Group social activities		
Yes	1.00	<0.0001
No	2.62 (1.63–4.23)	

*STARU* screening tool for assessing risk of undernutrition, *OR* odds ratio, *95 % IC* 95 % confidence interval; OR >1 indicate higher odds of poor SPH; OR <1 indicate lower odds of poor SPH; Estimates are based on *n* = 632 participants due to missing values; Model diagnostics AUC = 0.810; Hosmer-Lemeshow-*p*-value = 0.152; *R*-square = 0.169; Max-Rescaled 0.318

## Discussion

This study aimed to assess the role of various individual, social and contextual factors in the assessment of SPH in independent older people without cognitive impairment. For this purpose, individuals were considered independent if they had good cognitive status and BADL function. In past research [11], people with cognitive impairment have also been excluded, given their level of deterioration could interfere with their ability to provide valid answers. Inability to perform daily life activities was also an exclusion criteria applied in a previous older people study [11]. Therefore, the approach followed in the present study can provide further research information on which other factors besides autonomy are associated with SPH.

The multivariate model that best explained poor SPH in our data, after adjusting for age and sex, included the following variables: polypharmacy, having sensory impairment, bad sleep quality, bad nutrition pattern and not engaging in cognitively stimulating activities or group social activities. These results are consistent with the complex definition of AHA [3, 38, 39]. The presented model includes factors related to individual health status, like polypharmacy, clearly related to the presence of chronic diseases. Also three elements rarely relevant in younger populations, namely, sensory problems, sleep quality and nutrition patterns [38–40]. Additionally, our model includes two variables related to enjoyable activities: cognitively stimulating activities and group social activities. As far as cognitively stimulating activities are concerned, reading may be associated with educational attainment, but not listening to the radio. This finding may indicate the importance of maintaining mental vitality into late adulthood as a component of healthy ageing. A WHO report of active ageing [39] encourages older people to keep learning to maintain cognitive capacities. Other relevant aspects associated with a satisfactory cognitive ageing are the absence of stress in older people, found to be related to a lower deleterious impact in the brain [41], and brain reserve, where higher mental activities have been found to be associated with less cognitive decline [42]. Group social activities are a multicomponent variable that reflects a good level of interest in socialising and maintaining a social network. Both cognitively stimulating activities and group social activities are factors that may contribute to improving quality of life of older people, being the latter also important to enhancing social connectedness [43]*.* Previous works have also examined the relation between social activities, social participation or social integration and SPH [11, 13]. Although different social activities have been studied, such as, participation in recreational activities at a public hall or community association for the aged [11], in a hobby association [11], in educational and cultural activities [13], in sports or physical activities with other people, in family/friendship activities outside the household [13], or going to a social centre for the elderly [12]; in all cases, participants had better SPH than non-participants.

Age, sex, education, income, chronic diseases, functional status and mental health have also been found to be linked to SPH in previous studies [10–12]. In our data of functionally independent subjects, all other factors but sex were associated with SPH in the univariate analysis stage. The lack of association with sex is completely unexpected and is probably due to the selection criteria applied, in particular, the exclusion of dependent individuals, with dependence being more common among women. However, sex was not a predictor of SPH in a previous study of older Thai people [10].

On the other hand, our finding that good night sleep is related to a better SPH is consistent with a cohort study of non-institutionalized Spanish adults aged 60 years or more where extreme sleep durations (≤5 or ≥ 10 h) were associated with a poorer health-related quality of life [44]. Sleep is a vital physiological process and the mechanisms implicated in its duration and quality are complex and multidimensional [45]. Several changes are seen in older people, as aged individuals, are more prone to wake up at earlier times or experienced an involuntary reduction of their total sleep duration [46]. Finally, disturbances in the circadian rhythm have been associated with a worse health [47]. All the above reveals the importance of maintaining adequate sleep patterns in this population group.

Sensory-related abilities are also found to be related to SPH. This is in concordance with a study of non-disabled older people living alone [11], where problems in visual, hearing and chewing abilities were associated with poor SPH. One of the challenges of an ageing population according to WHO are precisely vision and hearing losses as well as oral health [39]. The WHO urges countries to develop policies and programmes to prevent visual and hearing impairment and promote good oral health habits [39]. Similarly, the development of guidelines promoting an adequate nutrition is a need to solve eating problems, understanding as such both under nutrition and excess energy intakes [39].

Another difference with other published studies [14, 15] is the lack of associations with neighbourhood characteristics (adequate community services and proximity to a number of facilities), either in the univariate (except for proximity to supermarket) or in the multivariate analysis. One possible explanation is that people with poor SPH may have a different perception of their neighbourhood environment than those in good health [48] and may not even perceive these problems as they go out less frequently. It is also likely that, if individuals live in the same neighbourhood for a long time, they get used to the environment. Another plausible justification may be that the measure created to analyse this factor was not suitable for use in older people. Finally, it is also important to consider that the questions used to assess community services were based on WHO age friendly city criteria [35] and were not part of an specific or validated questionnaire. Therefore, further research will be conducted exploring this issue and, perhaps, new tools should be developed to explore elderly peoples’ perception of their communities.

Several strengths of our study deserve to be highlighted. Firstly, the study was based on a large sample of community-dwelling older people and we have investigated the association between SPH and multiple dimensions of health and living conditions, using a wide range of individual, social and contextual variables*.* Another important characteristic of this study was the collaborative participation of the multidisciplinary expert panel. They reviewed and commented on the draft of the questionnaire and helped with the development of the final version, from a comprehensive multifactorial perspective.

On the other hand, there are limitations that should be recognised. The study design was cross-sectional, and hence, it is not possible to establish cause-effect relationships. Nonetheless, as stated by Giron et al. [19], this type of research is necessary to help us understand factors that determine or alter health. Moreover, the battery of questions developed is extensive (145 items that were responded in approximately 60 min) and may explain why individuals who agreed to participate tended to have a relatively good functional status. Another limitation of this study is that the data were collected via face-to-face interviews by 20 interviewers. So, although interviewers were trained, there is always a possibility that the data collection technique varied, as was explained in the study of Tajvar et al. [49]. Nevertheless, face-to-face interviews are the recommended way to collect information in the case of long questionnaires, and generally obtain higher response rates than postal or telephone interviews [50]. Even though information of chronic conditions was self-reported by the individuals and thus may affect the accuracy of information, it is an approach used in similar previous studies [10, 11]. It is also important to discuss about the representativeness of the sample. The original sample (800 individuals) was representative of the Gipuzkoa elderly population according to sex and age. The exclusion of non-independent and cognitively impaired individuals did not cause major alterations to the percentage of the considered variables, as in the finally included sample (634 individuals) all categories were between 1 and 5 % of the original sample. Finally, missing data was not a particular problem in the current study but for the income level variable, which 22 % of the sample did not reply. It is likely that full information on this very variable would have allowed for further explorations.

## Conclusions

In conclusion, several different factors related to health and social variables account for the SPH of older people besides autonomy. Among them, the study found that drugs taken per day, sensory-related abilities, sleep quality, nutrition and leisure activities were the most strongly associated with SPH in a functionally independent elder population. In this sense, our results confirm the multidimensional nature of SPH and are consistent with those of other studies. Our work highlights the need for a thorough assessment of factors associated with SPH in older adults, this being essential for developing health-related programmes that promote active and healthy ageing.

## Abbreviations

AHA: active and healthy ageing
BADL: basic activities of daily living
BI: Barthel index
CI: confidence intervals
Duke-UNC FSSQ: Duke-UNC functional social support questionnaire
EQ-5D: EuroQol 5D questionnaire
GDS: geriatric depression scale
LS: Lawton instrumental activities of daily living scale
LSNS: Lubben social network scale
OR: odds ratios
SD: standard deviation
SPH: self-perceived health
SPMSQ: short portable mental status questionnaire of Pfeiffer
STARU: screening tool for assessing risk of undernutrition
STS: social trust scale
WHO: World Health Organization
